# Fast and Filtration-Free Method to Prepare Lactic Acid-Modified Cellulose Nanopaper

**DOI:** 10.1021/acsomega.1c02328

**Published:** 2021-07-15

**Authors:** Jatin Sethi, Henrikki Liimatainen, Juho Antti Sirviö

**Affiliations:** † Fibre and Particle Engineering Research Unit, University of Oulu, P.O. Box 4300, 90014 Oulu, Finland; ‡ Fibre Technology Division, KTH Royal Institute of Technology, Teknikringen 58, SE-100 44 Stockholm, Sweden

## Abstract

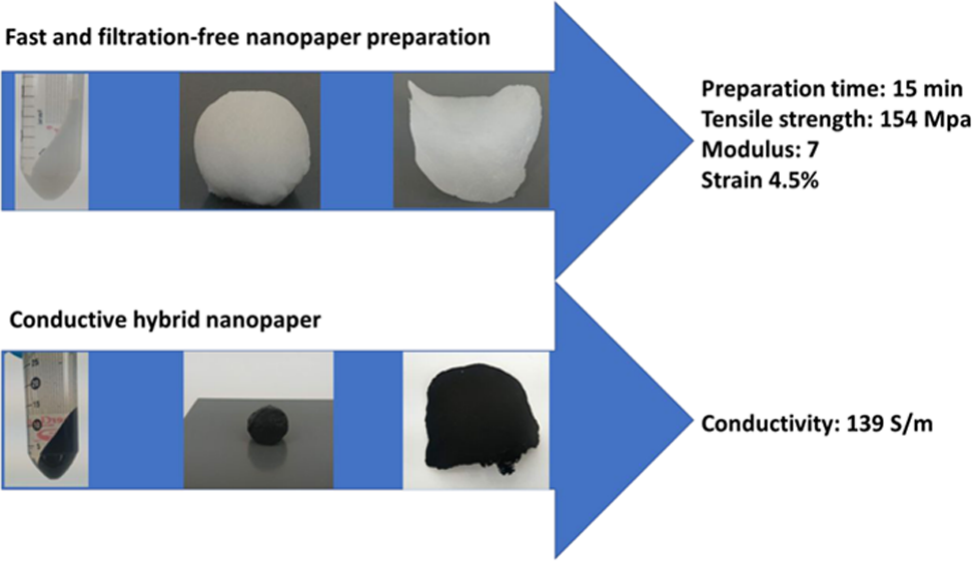

Dewatering in the preparation of cellulose nanopapers can take up to a few hours, which is a notable bottleneck in the commercialization of nanopapers. As a solution, we report a filtration-free method that is capable of preparing lactic acid-modified cellulose nanopapers within a few minutes. The bleached cellulose nanofibers (CNFs), obtained using a Masuko grinder, were functionalized by sonication-assisted lactic acid modification and centrifuged at 14 000 rpm to achieve a doughlike, concentrated mass. The concentrated CNFs were rolled into a wet sheet and dried in a vacuum drier to obtain nanopapers. The nanopaper preparation time was 10 min, which is significantly faster than the earlier time period reported in the literature (up to a few hours of preparation time). The mechanical properties of nanopaper were comparable to the previous values reported for nanopapers. In addition, the method was successfully used to prepare highly conductive functional nanopapers containing carboxylated multiwalled carbon nanotubes.

## 1 Introduction

In the past few decades, plastics have been the most popular choice for packaging materials. They have high barriers to oxygen, and they are highly transparent. Additionally, they are lightweight, cheap, and easy to process. Unfortunately, plastic is nonbiodegradable, and therefore, it is impossible to get rid of them in an eco-friendly way. Used plastic pollutes the oceans and the lands, and when burned, contributes to the greenhouse effect, which adds to the global warming. In recent times, there has been an accelerated attempt to replace plastic-based packaging with a natural crop-based product, one that is easy to manufacture such as paper but has comparable properties to that of plastic.

Conventional paper is prepared from cellulose macrofibres, which leads to inefficient packing and a highly porous structure. This is the major cause of poor barrier properties and inferior mechanical properties. This problem is solved by making papers with cellulose nanofibers (CNFs). They have good oxygen barriers, are transparent, and have phenomenal mechanical properties.^1,2^ However, they are not yet commercially available; presumably, due to the very slow dewatering process of aqueous cellulose nanofiber (CNF) suspensions.

Cellulose nanofibers have a huge surface area and thus have a large amount of pendant hydroxyl groups, which binds a huge amount of water. Conventional cellulose macrofibres drain in few seconds, but cellulose nanofibers can take up to few hours to drain.^3^ Thus, fast drainage or water removal from CNF suspensions is of paramount importance. Only a handful of articles are available that have addressed the improvement of the draining time of nanopapers,^4−6^ and the industrial adaptability of many of the suggested approaches can be questioned.

Apart from a long preparation time, drainage (i.e., removal of free water from the nanopaper in a vacuum) has inherent drawbacks, such as high energy consumption and the partial retention of nanocellulose in the wet film. The nanopapers are typically prepared by filtering a dilute suspension through a fine-sized polymeric membrane (with a submicron pore size)—which not only allows water but also nanosized particles to partially pass through, with the yield loss being as high as 40 wt %.^4^

Additionally, this slow preparation time of nanopapers is also slowing the advancement of nanopapers in the field of functional materials. MWCNT-based conductive nanopapers, for example, are excellent material for electrodes of supercapacitors. However, with current methods, they cannot be prepared at a pace where they can be commercialized. Furthermore, adding multiwalled carbon nanotubes (MWCNTs) to CNF suspension will add to the problem of low retention as they are relatively smaller than CNFs and passes through draining membranes. MWCNT, unlike CNFs, are really expensive and losing them in filtrate will add significantly to production cost. Yoon et al. found a novel way to increase the retention by dipping finished CNF nanopaper in MWCNT dispersion instead of draining; however, it adds 24 h to the preparation time.^7^ Therefore, a method to swiftly prepare nanopaper will help the field of advanced nanocellulosic material immensely.

In this study, we propose an alternative approach: a fast and filtration-free method for the fabrication of CNF nanopapers. We earlier reported that sonication-assisted lactic acid (LA) modification reduces water retention in nanopapers, shortening the draining time by 75%.^8,9^ Here, the LA modification of CNFs was used in combination with centrifugation treatment to get rid of water, entirely omitting the need for vacuum filtration and thus significantly reducing the preparation time. Dried nanopapers were ready to use in 10 min (15 min with LA modification). To the best of our knowledge, such a short time for the nanopaper production has never been reported before. These nanopapers were characterized by their mechanical properties and morphology. In addition, conductive, functional nanopapers containing carboxylated multiwalled carbon nanotubes (MWCNTs) were prepared.

## 2 Results and Discussion

### 2.1 CNF–Water Interaction and LA Modification

**Figure 1:**
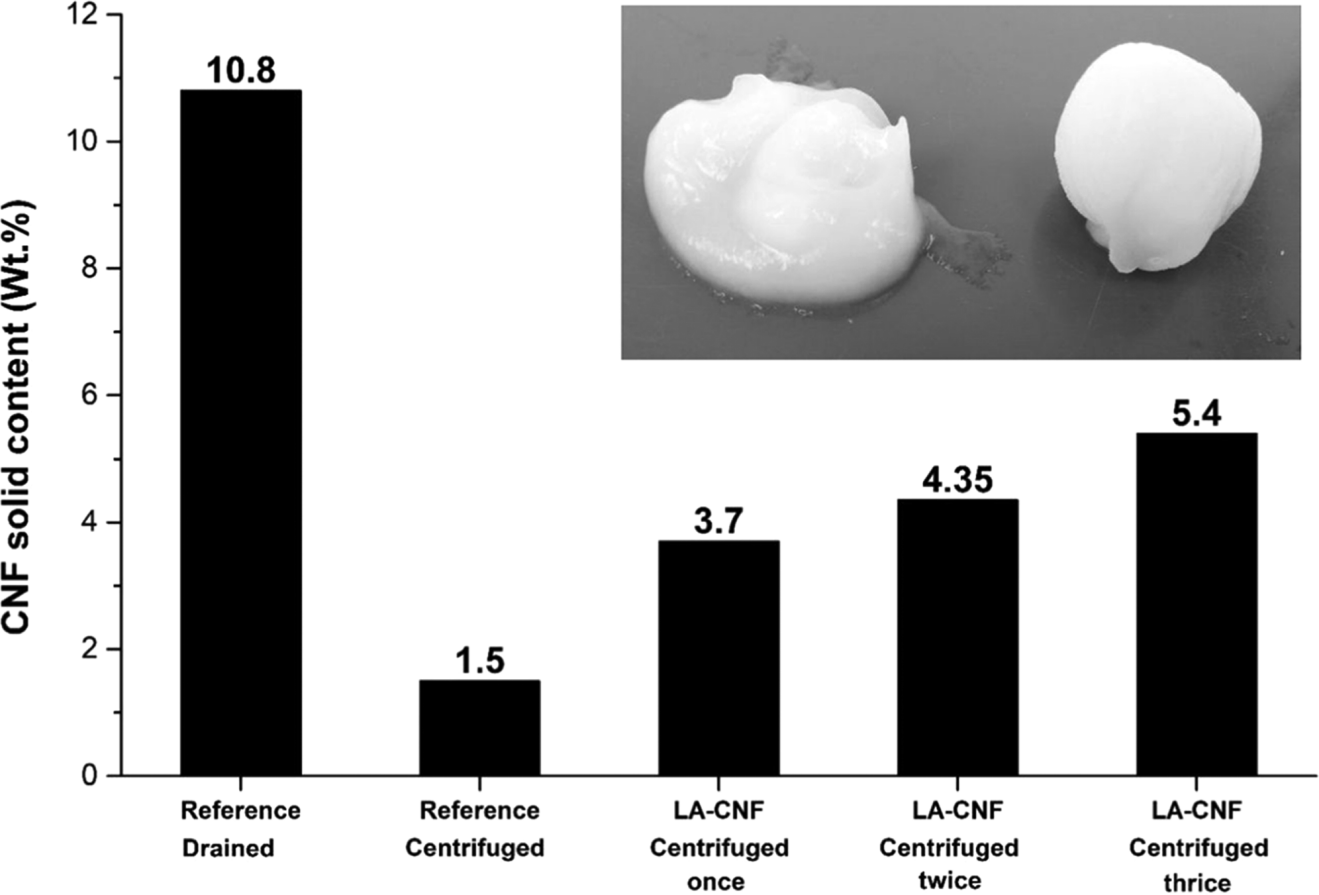
Dry matter content of unmodified and lactic acid-modified cellulose nanofiber suspensions after centrifugation and filtration (reference filtration) methods (Inset: unmodified and lactic acid-modified cellulose nanofiber gels after centrifugation).

Due to the low water retention tendency of LA-modified CNFs,^8^ water was rapidly released under centrifugal force from the dilute CNF suspension (Figure 4). The dry matter content of centrifuged reference (unmodified) and LA-modified CNF suspensions are presented in Figure 1. After centrifugation, reference (unmodified) CNFs had a solid content of 1.5 wt %, while LA-modified CNFs had a solid content of 3.7 wt % (an increase of 150%). Clearly, the water retention of CNFs was drastically reduced after LA modification. At 3.7 wt % solid content, LA-modified CNFs existed as a doughlike gel, while the reference (unmodified) CNF formed a flowable semifluid gel (Figure 1 inset). The self-sustaining mass of LA-modified CNFs could easily be shaped into a thin sheet by simple pressing and could be dried under a vacuum to obtain a uniform nanopaper. The water removal process using centrifugation required a relatively high centrifugal force (>12 000*g*), and it was experimentally found that a rotational speed lower than 10 000 rpm was not sufficient to obtain enough solid content. The centrifugation time of 5 min was enough to produce a doughlike CNF gel.

CNF nanopapers have commonly been produced using a vacuum filtration process, combined with oven-drying or hot-pressing. These methods are, however, time-consuming and require a processing time of a few hours.^3^ Moreover, a typical film-forming technique based on suspension casting is even slower and can take 5–6 days to results in a self-standing solid film.^3^ The shortest processing time that has been reported based on a vacuum filtration method using LA-modified CNFs is 30 min.^9^ A comparison of nanopaper preparation time from literature is enlisted in Table 1.

**Table 1:** Comparison of Draining Times and Total Processing Times of Different Nanopaper Production Methods

reference	dewatering time (min)	total preparation time (min)	CNF concentration (wt %)	volume (mL)	diameter, thickness	dewatering method
this study	5	15	0.3	200	80 mm, 60 μm	centrifugation
(9)	15	30	0.2	250	72 mm, 80 μm	vacuum filtration
(100)	45	60	0.3	80	72 mm,	vacuum filtration
(3)	45	150	0.2		72 mm, 55 μm	vacuum filtration
(4)	60	90	0.84	150	132 mm, 120 μm	vacuum filtration
(13)	120	150	0.2	250	72 mm, 80 μm	vacuum filtration
(101)	157	167	0.5			vacuum filtration

In the present filtration-free approach, we were able to complete the dewatering process within 5 min, which is the fastest reported value for CNFs (to the best of our knowledge). With LA modification, the total processing time from unmodified CNFs to finished nanopaper was 15 min (10 min from LA-modified CNF suspension), which is considerably faster than the value reported earlier. Water can be removed by centrifugation through the filtration-free method used in this study and nanopaper can be prepared at a different site from the drained gel. Centrifugation is commonly used for the separation of solids from liquids and is widely used in many industries such as wastewater processing industry, pharmaceutical industry, biotechnology industry, food processing industry, mining industry, and so on.^10^ It can be easily scaled up for processing of large volumes and does not have the limitations related to vacuum-assisted filtration-based systems.^11^

The unmodified nanocellulose retains a high amount of water because of its high content of free hydroxyl groups and its very large surface area.^12^ On the other hand, regular pulp fibers have a notable smaller surface area and the water removal time is manageable with the industrial draining setups. After the LA modification of CNFs, hydroxyl groups were replaced by more hydrophobic LA moieties.^9^ The effect of LA modification on the water holding capacity of the CNF was indicated by the water retention value (WRV), which showed a decrease in the WRV from 66 to 26% for LA-modified CNFs. The WRV further decreased to 17% after repeated centrifugation. The role of hydroxyl groups in water removal can also be observed from lignin-rich cellulose nanofibers, which are morphologically similar but devoid of hydroxyl groups (as lignin encapsulates the fibers). At same temperature and pressure, unmodified CNFs drain in 120 min while lignin-rich cellulose nanofibers drain in 15 min in a membrane filtration process.^13^

As a reference, vacuum filtration was used to test the efficiency of water removal from CNF suspension. The vacuum-drained wet CNF gel from unmodified CNFs had a solid content of 10% after 120 min of filtration (Figure 1). The CNF dried with centrifugation had a solid content of 5.4 wt % after 10 min of centrifugation, highlighting the swiftness of centrifugation in dewatering. However, optimization of the centrifugation process is still required to make the process more efficient.

We did not find any study that has reported the preparation of a large nanopaper as the size of nanopaper is restricted to the diameter of the draining setup, which is few centimeters wide. Therefore, we attempted of make a large nanopaper (A4 Size) using the filtration-free method. The drained LA-CNFs were rolled in a large sheet and dried on a 70 μm polyester cloth, and then dried in a melt press. Figure S1 (Supporting information) demonstrates that large nanopapers can be prepared by a filtration-free method; for comparison, a nanopaper drained using filtration setup is presented. The LA-modified CNFs were easily collected into a larger mass and rolled into sheets that were dried into nanopaper sheets. Therefore, the centrifuged CNF with a high solid can be further fed into the continuous sheet press to enable an industrially feasible process for the nanopaper production.

### 2.2 Morphology of Nanopapers

**Figure 2:**
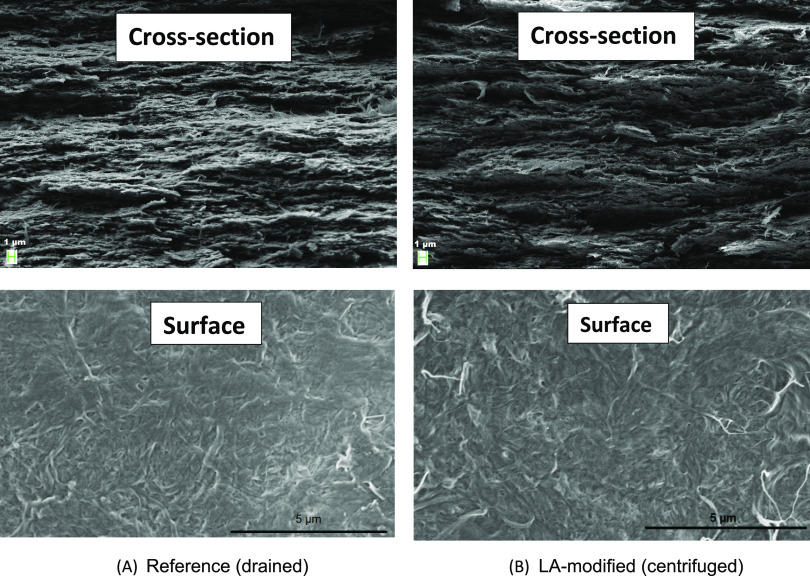
FESEM micrographs of fractured samples from tensile samples and planar surfaces of (A) reference nanopaper prepared from the unmodified pulp through filtration and (B) lactic acid-modified nanopaper from the filtration-free method.

The morphologies of the reference (vacuum-drained unmodified CNF) and the LA-modified CNF using the filtration-free process are presented in Figure 2. In both nanopapers, the CNFs were arranged in a planar layered fashion, and no visible differences in the structural distribution of the nanofibers were detectable. This layered arrangement is well known for nanopapers obtained from filtration,^8^ but was also noted here for the centrifuged nanopapers. During the filtration process, the nanofibers were finely arranged by concentration-induced floc aggregation into a thick and dense layer.^14^ As water drained, the colloidally stable nanocellulose reached a critical distance where the van der Waals force and the hydrogen bonding produced an aggregate. The aggregates, in turn, formed a nanopaper by a layer by layer deposition. This condensed morphology is a major reason for the excellent mechanical properties of the nanopapers. Interestingly, when the centrifuged CNF gel was pressed under a vacuum (Rapid Köthen equipment), the CNFs were also arranged in a layered structure. We expected no directional orientation, but, evidentially, nanocellulose prefers layered arrangement, and the reason for this is yet to be studied. Similarly, no evident difference was visible on the surface of the nanopapers. This similar morphology is the reason for the comparable mechanical properties of nanopapers obtained from vacuum filtration and is explained in the next section.

### 2.3 Mechanical Properties of the Nanopaper

The filtration-free nanopaper from the LA-modified CNF had a modulus, tensile strength, and strain of 7 GPa, 154 MPa, and 4.5%, respectively. When compared to values reported in the literature, the mechanical characteristics of nanopapers obtained from the centrifugal drying were similar or better (Table 2). The reference (control sample) prepared by draining of unmodified CNFs through a membrane had the modulus of 7.2 GPa and the tensile strength of 220 MPa. Though the modulus was similar, the tensile strength decreased in LA-modified nanopapers by 30%. This could be because of the presence of LA moieties at the interface, which likely reduced the hydrogen bonding between the CNFs.^14^

**Table 2:** Comparison of Mechanical Properties of Nanopapers Reported in the Literature

reference	method of preparation/equipment	raw material	modulus (GPa)	tensile strength (MPa)	strain (%)
this study	masuko grinder	softwood sulfite pulp	7	154	4.5
(8)	masuko grinder	softwood sulfite pulp	6.4	170	11
(3)	microfluidizer M-110EH	softwood sulfite pulp	10	178	6.3
(15)	microfluidizer M-110EH	softwood pulp	10	200	5
(16)	masuko grinder	waste paper pulp	6.7	135	
(17)	niro Soavi homogenizer	bleached almond shell	5.3	65	4.19
(18)	supermass collider MKCA6-3	bleached maize stalk	8.8	95	2.3
(19)	manton-Gaulin homogenizer	bleached softwood	6.5		
(19)	manton-Gaulin homogenizer	bleached softwood	6.3	91	
(20)	microfluidizer M-100 y	sulfite pulp	6	100	4.5
(21)	emulsiFlex-C5 Homogenizer	bleached Triodia pungens	3.2	84	18
(22)	masuko grinder	sulfate spruce pulp	9	130	6.5
(23)	masuko grinder	cellulose sludge	3.6	57	3.4
(17)	high-pressure homogenizer	bleached pulp from almond shell	5.62	63	2.9
(24)	homogenizer	bleached pulp	6.3		
(24)	microgrinder	bleached pulp	4.4		
(25)	high-pressure homogenization	hardwood pulp	3	70	2.3
(25)	high-pressure homogenization	softwood pulp	2.5	80	6
(26)	homogenizer	bleached tobacco stalk	5	180	
(27)	homogenizer	beech wood pulp	7.8	150	5
(28)	ultrafine friction grinding	bleached kraft bagasse pulp	6.1	110	
(28)	ultrafine friction grinding and homogenizer	bleached rice straw pulp	4.7	68	
(29)	homogenization	enzyme-treated sulfite-based softwood dissolving pulp	7.5	91	2.9
(30)	masuko grinder and high-pressure homogenizer	untreated bagasse pulp	6.1	110	

The mechanical properties can be further improved by polymerizing LA oligomers at a high temperature and pressure. Earlier, we have reported an increase of 30% in elastic modulus by polymerizing LA oligomers.^8^ Furthermore, nanopapers (after polymerizing of LA) were water-resistant^8^ and dimensionally more stable.^9^ However, polymerization under high temperature and pressure makes nanopaper brittle. Finally, it is worth mentioning that the nanocellulose used in this work was mechanically ground and no harmful modifications were done. The grinding was conducted at room temperature and in an aqueous medium, making this method completely green in nature. However, there is an underlying restriction regarding the use of green chemicals pertinent to a filtration-free method, which originates from the production of pulp from wood. The chemicals such as sodium sulfide and sodium hydroxide are used in delignification. However, we do not see any threat from aforementioned chemicals and pulping process currently as it is a closed process, which recycles all chemicals and leads close to zero emission.

### 2.4 MWCNT-Containing Functional Nanopapers

One of the most sought-after use of conductive nanocellulose paper is use in advanced electrical equipment as such papers can be useful in electromagnetic interference shielding, supercapacitors, and electronic circuits.^31^ Therefore, we tested this method to prepare MWCNT-reinforced nanopapers. We found out that this method can be used to prepare conductive nanopapers without any problems. Figure 5 presents the photographic images of the sample preparation. The flexible nanopapers with 10 wt % MWCNT were prepared in 10 min. They were electrically conductive, having the conductivity of 139 S/m, which is higher than previously reported for MWCNT nanopapers with 10 wt % or a higher MWCNT concentration (Table 3). This makes the filtration-free method a promising approach for the design of functionalized nanosheets. We would like to mention that there are preliminary results and a detailed study is under the planning stage.

**Table 3:** Comparison of Electrical Conductivity of Multiwalled Carbon Nanotube-Cellulose Nanofiber (MWCNT-CNF) Nanopapers (MWCNT Content of 10 wt % or Higher) in Literature

sample	conductivity (S/m)	CNT concentration (wt %)
this study	139	10
(31)	0.1	18
(32)	120	13.9
(33)	78	50
(34)	37.6	10

### 2.5 Upscaling of the Filtration-Free Process

**Figure 3:**
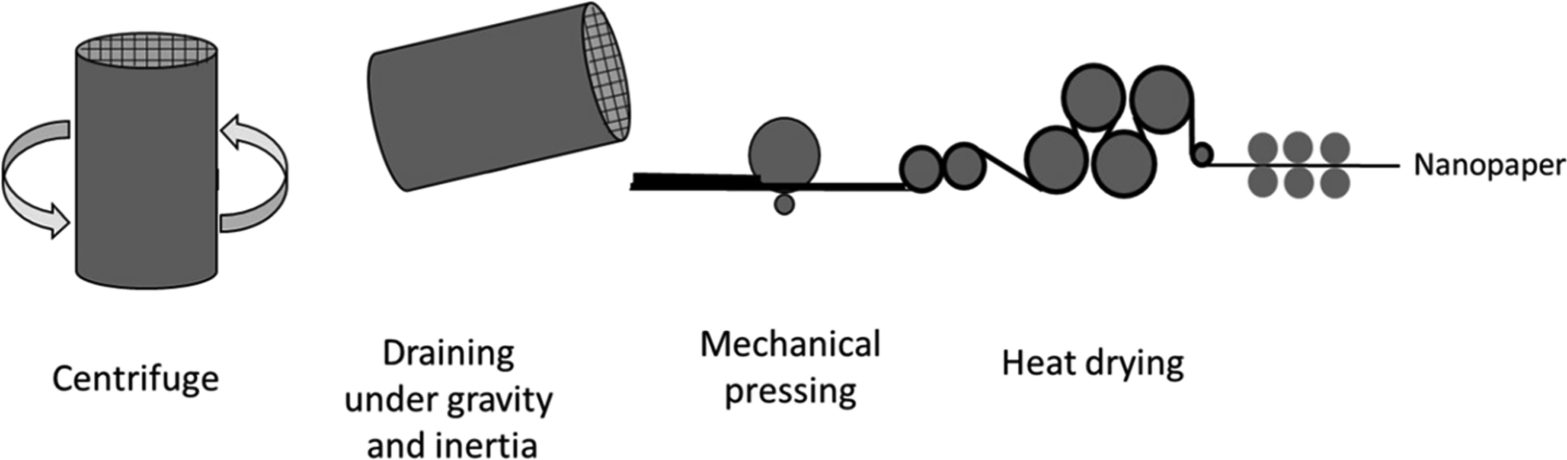
Upscaling of the filtration-free method to prepare a cellulose nanopaper from an LA-modified CNF.

Figure 3 presents a possible process flow of preparation of cellulose nanopaper from a filtration-free method on a large scale. After centrifugation, LA-modified CNF can be drained under gravity. The doughlike mass is then added to a papermaking line, which would need a finer mesh (wire) than the conventional papermaking process. In the lab experiments, a mesh with a pore size of 250 μm was sufficient. A coarser mesh can also be used, but an optimization is needed. The LA CNF is then passed through mechanical rollers, which serve two purposes: flatten the dough into sheets and remove extra water through mechanical pressing. The final stage is heat-drying through steam rollers, which evaporate the residual water to form a dry nanopaper. The parameters such as length and speed of the process and temperature of zones/rolls need to be optimized. It is likely that these parameters can be different when compared to the current papermaking standard. For example, current paper machines operate up to 2000 meters per minute; such high speeds are unlikely because of the limited capacity and speed of centrifugation. Therefore, further research is required related to the upscaling.

## 3 Materials and Methods

CNFs were prepared by grinding the bleached softwood sulfite pulp (Stora Enso, Finland) with a Masuko supermass collider (MKCA6-2 JCE; Masuko Sangyo, Japan). The pulp was diluted to the consistency of 1.6 wt % and repeatedly fed into the grinder. The distance between the grinding stones was gradually decreased from −20 to −40 μm, −60 and −90 μm, and the pulp was repeatedly passed through the grinder two, three, four, five, and seven times, respectively.

Carboxylated MWCNT (purity >98%) powder was purchased from TimesNano (China). The average length and diameter of the MWCNT were 20 μm and 8 nm, respectively. l-(+)-Lactic acid (80%) and sodium dodecyl sulfate were purchased from Sigma-Aldrich (Finland).

### 3.1 Preparation of LA-Modified Nanopaper

**Figure 4:**
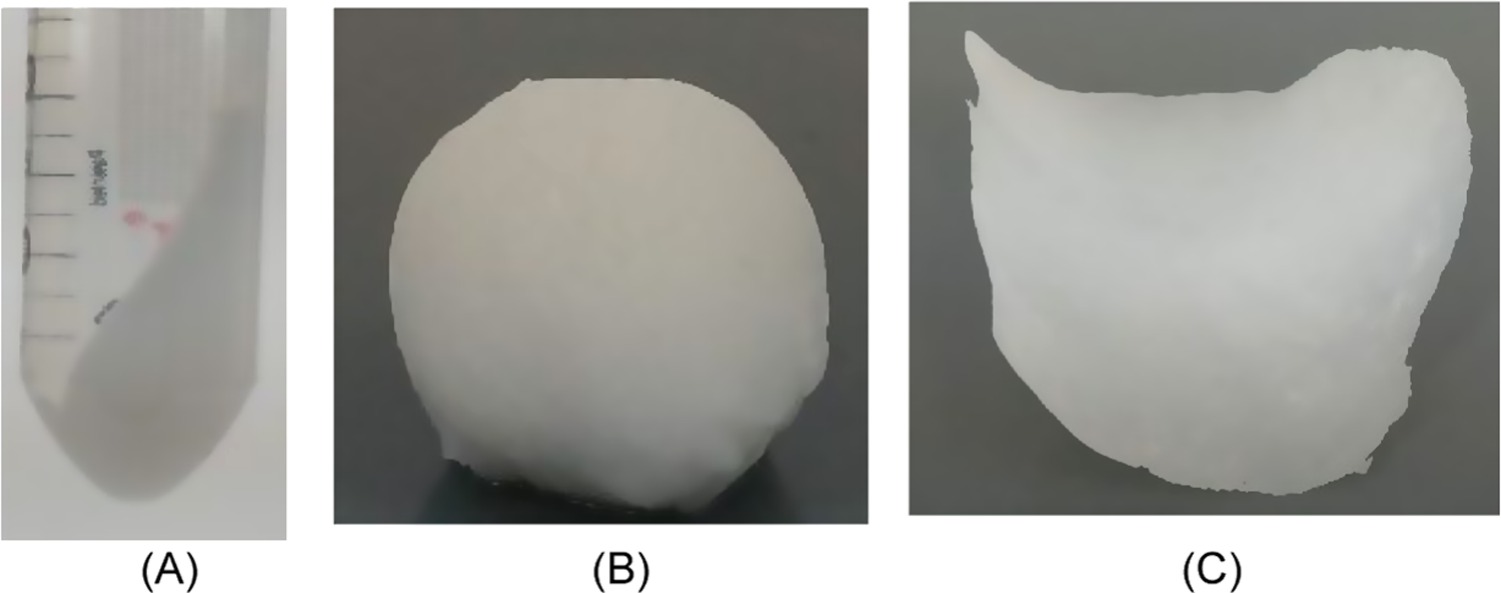
Preparation of lactic acid-modified cellulose nanopaper using a filtration-free method. (A) Separated cellulose nanofiber (CNF) mass after centrifugation, (B) doughlike mass of lactic acid-modified CNF after centrifugation, and (C) dried CNF nanopaper.

CNFs were modified by LA in the presence of sonication by adapting the method reported earlier.^8^ In short, the CNF suspension was diluted to the concentration of 0.3 wt %, and LA was added to the suspension so that the amount of dry CNF was equal to the amount of LA. The CNF-LA water suspension was stirred with the Ultra-Turrax mixer at 10 000 rpm for 5 min. Thereafter, the CNF-LA suspension was sonicated until the sonication energy imparted was 300 J/mL with the Hielscher UP 400s probe-type sonicator equipped with a titanium tip. LA-modified CNF (50 mL) was added to four polypropylene tubes and centrifuged at 14 000 rpm for 2 min (Figure 4A). The rotational speed was selected on the basis of a pretrial. The supernatant was drained under gravity and precipitated CNFs from the four tubes were recollected into a single test tube and recentrifuged at 14 000 rpm for 3 min. This process resulted in a doughlike material, as shown in Figure 4B. The concentrated CNF suspension was rolled into a thin sheet with the aid of cylindrical beaker, and the sheet was dried under a Rapid Köthen drier in a vacuum at 90 kPa (for 5 min).

### 3.2 Preparation of MWCNT-Reinforced Nanopapers

**Figure 5:**
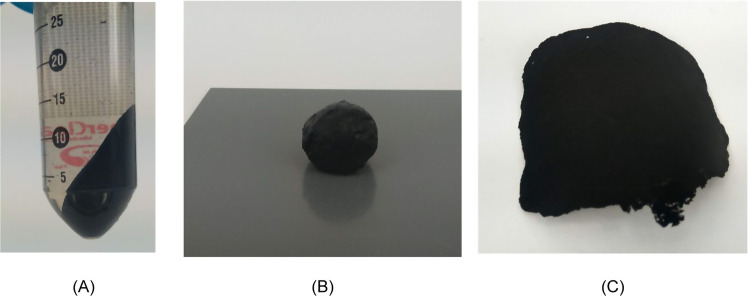
Preparation of multiwalled carbon nanotube (MWCNT)-containing conductive nanopaper. (A) Suspension containing cellulose nanofiber (CNF) and MWCNT after centrifugation, (B) doughlike mass-containing CNFs and MWCNTs, and (C) dried MWCNT-CNF nanopaper.

MWCNTs (0.3 wt %) were added to an aqueous sodium dodecyl sulfate solution, and the mixture was sonicated with the Hielscher UP 400s probe-type sonicator until the sonication energy was 5000 J/mL. Then, the mixture was added to an LA-CNF suspension (0.3 wt %) and sonicated. The concentration of MWCNT was 10 wt % of dry CNF content in the suspension. Nanopapers containing MWCNT were fabricated as described below (Figure 5).

### 3.3 Mechanical Testing of Nanopapers

A ZwickRoell universal testing machine was used to characterize the mechanical properties of nanopapers. Rectangular strips (50 mm × 5 mm) were cut from nanopapers with scissors and stored in ambient conditions (23 °C and 55% RH) for at least 48 h before testing. Samples were fastened to grips (20 mm apart) and stretched using a 1 kN load cell at a crosshead speed of 5 mm/min. The elastic modulus was determined by calculating the slope of the stress–strain curve in the linear region, and the yield strength was determined as the point of intersection of the stress–strain curve and offset line originating from a 0.2% coordinate.

### 3.4 Microstructure of Nanopapers

Scanning electron microscopy images of a cross-section of nanopapers (from tensile testing) were obtained using a Zeiss Zigma HD VP (Germany) electron microscope at an acceleration voltage of 5 kV. Prior to the measurement, samples were sputter-coated with platinum for 30 s using one coating cycle.

### 3.5 Electrical Conductivity

Samples were cut in a square shape of 1 cm × 1 cm, and conductive carbon paint was applied to the end. A digital multimeter was used to measure the resistance (*R*) across the square. The conductivity was calculated—as shown in eq 1, where σ, *L*, *w*, and *d* are conductivity, length, width, and thickness of the sample, respectively.
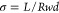
1

## 4 Conclusions

In this study, a rapid filtration-free method for the preparation of lactic acid-modified cellulose nanopapers was introduced. The total time of preparation was 15 min (5 min modification of CNFs, 5 min dewatering, and 5 min drying), which is far better than the dewatering and preparation time of nanopaper that have been reported in the literature. CNFs were modified by LA using an eco-friendly sustainable method based on sonochemistry. Centrifugation was used to remove water, and the remaining CNF mass, which had a doughlike consistency, was rolled into a sheet and dried in a vacuum dryer to get a LA-modified nanopaper. The nanopapers had a modulus of 7 GPa and tensile strength of 154 MPa, with an elongation of 4.5%. This method can be used to prepare large nanopapers, as well as functional nanopapers such as and MWCNT-CNF nanopapers. The conductivity of the MWCNT-CNF nanopaper was 139 S/m. The process is water-based, quick to apply, and results in 100% biobased material, which makes this process industrially adaptable.

## Supporting Information Available

Photographic images of nanopaper, FESEM of cross-section of modified nanopaper, and the stress–strain curves of reference and modified nanopapers (PDF)
