# Supplementary material for: Fast and Filtration-Free Method to Prepare Lactic Acid-Modified Cellulose Nanopaper
Source: ACS Omega. 2021 Jul 15;6(29):19038–44. doi: 10.1021/acsomega.1c02328 (PMC8320081; doi:10.1021/acsomega.1c02328)
Supplement: Supplementary file 1 — ao1c02328_si_001.pdf [file ao1c02328_si_001.pdf]

Fast and filtration-free method to prepare lactic acid modified cellulose nanopaper

Jatin Sethi[1,2], Henrikki Liimatainen[1], and Juho Antti Sirviö*[1]

1. Fiber and Particle Engineering Research Unit, University of Oulu, P.O. Box 4300, 90014 Oulu, Finland

2. Fibre Technology Division, KTH Royal Institute of Technology, Teknikringen 58, SE-100 44 Stockholm, Sweden

*Corresponding author: Juho Antti Sirviö, juho.sirvio@oulu.fi

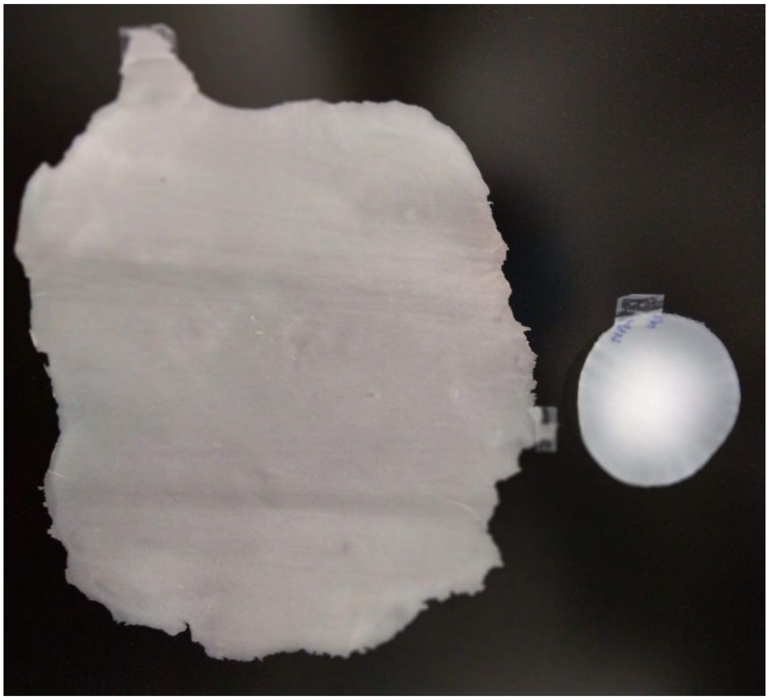

Figure S1 Large nanopaper prepared by filtration-free method (left); for comparison, drained nanopaper (right)

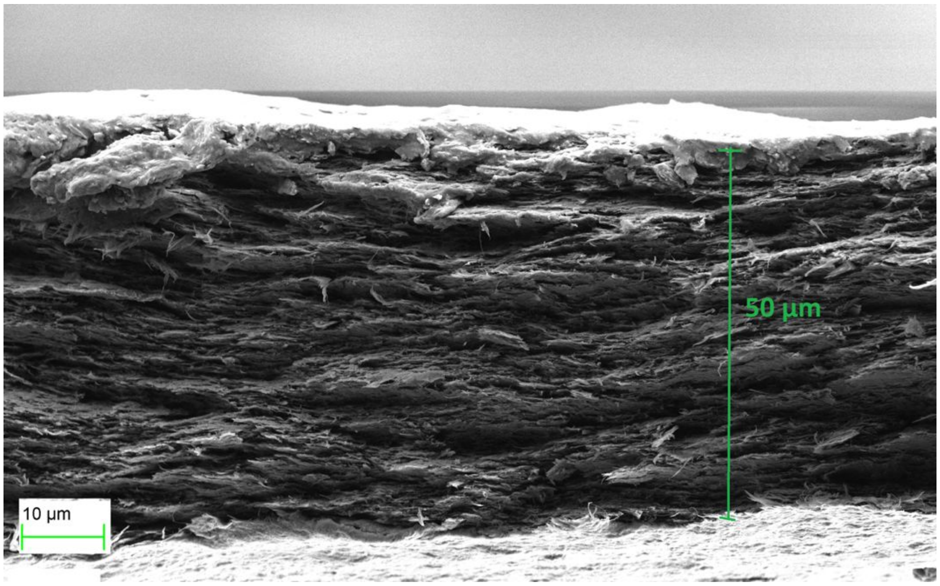

Figure S2 FESEM image of cross-section of nanopaper prepared from centrifugation

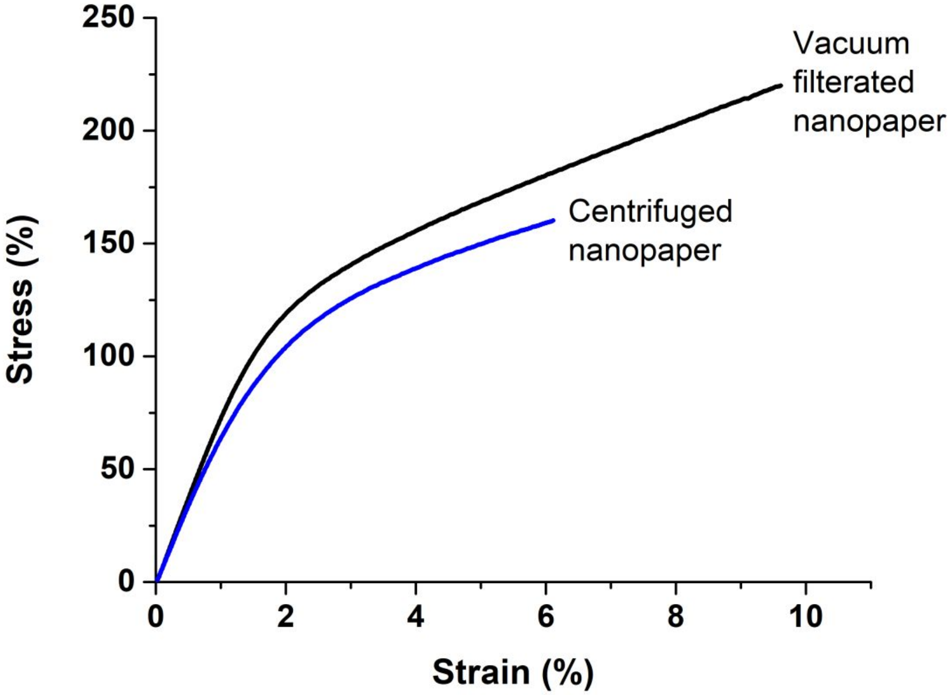

Figure S3 Stress-strain curves of centrifuged and filtered nanopapers
